# Congenital cystic adenomatoid malformation of the lung associated with bronchial atresia involving a different lobe in an adult patient: a case report

**DOI:** 10.1186/1752-1947-4-164

**Published:** 2010-05-28

**Authors:** Valerio DiScioscio, Paola Feraco, Alberto Bazzocchi, Rayka Femia, Chiara Romeo, Luca Fasano, Angela M Pacilli, Maurizio Zompatori

**Affiliations:** 1 Imaging Section, Department of Radiologic and Histocytopathologic Sciences, University of Bologna, S. Orsola--Malpighi Hospital, Via Massarenti 9, 40100 Bologna, Italy

## Abstract

**Introduction:**

Congenital cystic adenomatoid malformation of the lung is an uncommon cause of respiratory distress in neonates and babies. The disorder is usually diagnosed in the neonatal period and the first two years of life. This anomaly has been described in association with bronchopulmonary sequestration, extralobar intra-abdominal sequestration or bronchial atresia in live and stillborn babies. It is rarely encountered in adults, in whom the diagnosis is made incidentally from mass lesion features seen on chest radiographs. The oldest patients recorded with this malformation have been about 35 years old, and only 10% of primary diagnoses are made after the first year of life. Delayed diagnosis can be related to infection or serendipitous discovery.

**Case presentation:**

We describe the radiological findings of a 34-year-old Caucasian woman with a clinical history of recurrent pneumonia, intermittent anterior pleuritic chest pain and haemoptysis. Congenital cystic adenomatoid malformation of the lung associated with bronchial atresia involving a different lobe was discovered.

**Conclusion:**

Although rare in adults, congenital cystic adenomatoid malformation should be suspected in adult patients who suffer from recurrent or persistent non-productive coughs. The discovery of an association of congenital cystic adenomatoid malformation with bronchial atresia in adulthood is rare but possible, even in different lobes.

## Introduction

Congenital cystic adenomatoid malformation (CCAM) of the lung is a rare congenital pulmonary developmental malformation, found in terminal respiratory structures. It represents 25% of all congenital lung abnormalities and is characterized by a multicystic mass of pulmonary tissue with an abnormal proliferation of the bronchial structure [1]. About 46 cases of CCAM diagnosed in adulthood have been reported in the English literature up to now but none in association with bronchial atresia (BA) involving a different lobe [2].

We report a case of CCAM associated with BA discovered in an adult symptomatic patient and we describe the clinical features and radiological findings.

## Case presentation

A 34-year-old Caucasian woman with a clinical history of recurrent pneumonia and intermittent anterior pleuritic chest pain without risk factors, was hospitalized for hemoptysis. Pulmonary function tests were performed and revealed only a mild obstruction of the small airways. Diffusing capacity of the lung for carbon monoxide (DLCO) and pulmonary volumes were within the normal ranges.

**Figure 1:**
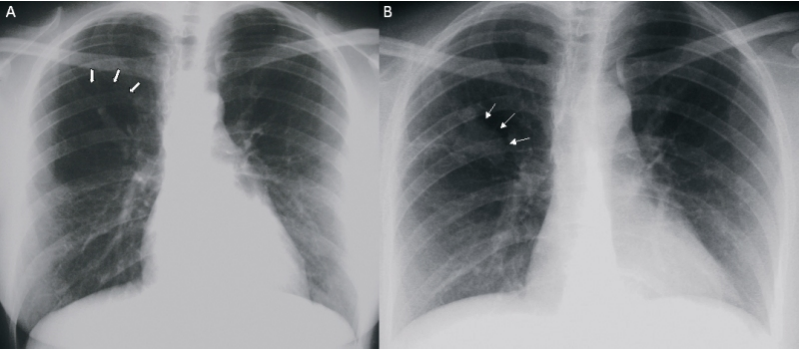
**(a) Posteroanterior chest X-ray showing an area of over-inflation and parenchymal oligemia downstream at the right upper lobe (thick arrows)**. (b) Posteroanterior chest X-ray showing right parahilar superimposed opacity (thin arrows).

**Figure 2:**
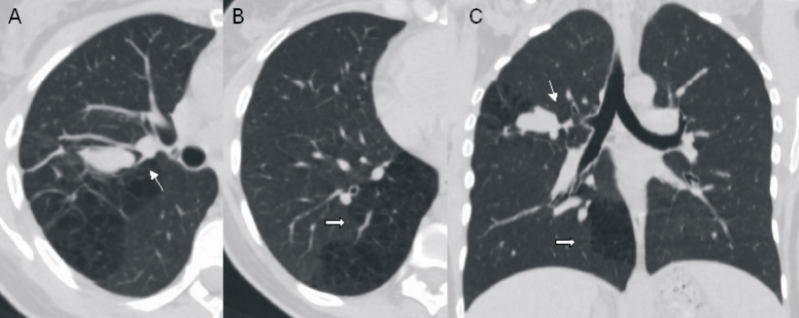
**Multi-detector computed tomography scan**. (a) Axial image shows a mucus-like density opacity at the right upper lobe. The bronchial branch for the right upper lobe is not identifiable. (b) Axial image of the right lower lobe allows the characterization of a malformed multicystic area of the lung parenchyma. (c) Malformative features in minimum intensity projection (minIP) coronal reconstruction (bronchial atresia: thin arrow; congenital cystic adenomatoid malformation: thick arrow).

**Figure 3:**
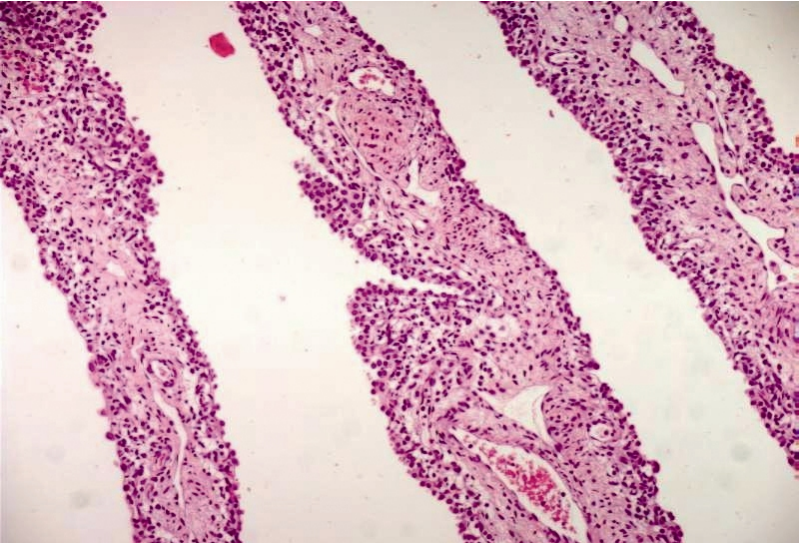
**Congenital cystic adenomatoid malformation**. The cystic wall lined by pseudostratified columnar epithelium (hematoxylin and eosin, 200×).

Chest radiography was performed at admission and showed an oval opacity at the bronchial branch for the right upper lobe with an area of parenchymal oligemia downstream, which had not been present in previous examinations (Figure 1). A multi-detector computed tomography (MDCT) scan of the lung was performed before and after intravenous non-ionic contrast medium administration and confirmed the over-inflation of the posterior segment of the right upper lobe with an atretic segmental bronchus, partially filled with mucus. Bronchoalveolar lavage, sputum and bronchial aspirate were negative for malignancies. Fiber-optic bronchoscopy confirmed stenosis of the bronchus of the posterior segment of the right upper lobe, and a diagnosis of BA was established. Symptoms and clinical history were then attributed to the discovered BA. High resolution computed tomography (HRCT) allowed this lesion to be better characterized, and another area of over-inflation with multiple air cysts connected to the segmental bronchus and well demarcated from normal lung parenchyma was detected in the apical segment of the right lower lobe. These findings were characteristic of CCAM type I (Figure 2) and the diagnosis was confirmed by biopsy (Figure 3).

Three weeks after treatment with antibiotics, chest radiography was repeated and this revealed the persistence of the upper lobe opacity, due to the mucus in the atretic bronchus that had not been washed.

Our patient is currently being monitored through regular follow-up exams.

## Discussion

Congenital cystic adenomatoid malformation has been diagnosed in association with other congenital lung malformations, such as bronchopulmonary sequestration, extralobar intra-abdominal sequestration or BA in live and stillborn babies, with involvement of the same lobe [3]. Depending primarily upon the volume of the lung affected, the abnormality may present at birth, or most commonly, in the neonatal period, when progressive air trapping in the malformed lung leads to respiratory distress. Only 10% of cases present after the first year of life [1,4] and only rarely is the presentation of CCAM delayed until adulthood; the oldest patients recorded with this malformation having been about 35 years old. Delayed diagnosis can be related to infection or serendipitous discovery. Although rare in adults, CCAM should be suspected in those adult patients who suffer from a recurrent or persistent non-productive cough. Clinical presentation in older patients is characterized by recurrent pulmonary infections, pneumothorax, hemoptysis, mycetoma or bronchioloalveolar carcinoma [5]. Due to its rarity, it is seldom suspected and adult physicians are not familiar with its clinical and radiological findings. Chest radiographs can suggest a localized patchy density, namely a cystic mass; but MDCT best demonstrates the cystic and solid components while ruling out bronchiectasis or a major bronchial obstruction.

The prognosis of CCAM presenting in adulthood depends on its pathological features, and the potential for malignant transformation [6]. Due to the paucity of reported cases, treatment guidelines have not been formulated. However, most experts recommend surgical resection to confirm the diagnosis and reduce the risk of infection or malignant transformation (bronchioloalveolar carcinoma).

Bronchial atresia and CCAM usually involve the same lobe and, although it has been stated that congenital cysts of the lung are due to abnormal bronchial development, the exact embryological and physiopathological mechanism is uncertain [7]. We have only observed coexisting BA and CCAM in different lobes in a single case and cannot give definitive information relevant to the pathogenetic models and theories. However, these findings suggest new insights into the pathogenesis of congenital malformations of the lung. Moreover the presence of BA in a different lobe limits the surgical approach, and indicates the possible need for a second operation if there are unbroken complications.

## Conclusion

The evaluation of cystic or multicystic lung disease in adults requires the consideration of a differential diagnosis and the investigation of acquired lesions, such as lung abscesses, cavitary neoplasms or inflammatory masses, bullous diseases, bronchiectases and post-inflammatory pneumatoceles. Clinical and histological correlations are essential in establishing a diagnosis but radiological studies can be definitive.

This is the first report of a diagnosis in adulthood of BA and CCAM with the involvement of different lobes.

## Abbreviations

CCAM: congenital cystic adenomatoid malformation; BA: bronchial atresia; MDCT: multi-detector computed tomography; HRCT: high resolution computed tomography; DLCO: carbon monoxide diffusing capacity.

## Consent

Written informed consent was obtained from the patient for publication of this case report and accompanying images. A copy of the written consent is available for review by the Editor-in-Chief of this journal.

## Competing interests

The authors declare that they have no competing interests.

## Authors' contributions

VDS, PF, AB, RF, CR, LF, AMP and MZ were involved in collecting the data and drafting the manuscript. All authors read and approved the final manuscript.
